# Can Personality Factors and Body Esteem Predict Imagery Ability in Dancers?

**DOI:** 10.3390/sports7060131

**Published:** 2019-05-29

**Authors:** Dagmara Budnik-Przybylska, Maria Kaźmierczak, Jacek Przybylski, Maurizio Bertollo

**Affiliations:** 1 Institute of Psychology, University of Gdansk, Gdansk 80-309, Poland; dagmara.budnik@ug.edu.pl (D.B.-P.); psymk@ug.edu.pl (M.K.); psyjp@ug.edu.pl (J.P.); 2 Department of Medicine and Aging Sciences, University “G. d’Annunzio” of Chieti-Pescara, Chieti 66100, Italy; m.bertollo@unich.it

**Keywords:** imagery, individual differences, body esteem, personality, sport

## Abstract

Dancing is mainly regarded as a form of art, which has been linked to the expression of emotions. Imagery is a well-known technique for enhancing performance. Additionally, specific personality traits are likely to facilitate performance. In the dancer’s performance, regarding the body as a tool is crucial. The following study examines personality and perceived body esteem as predictors of imagery ability in professional dancers. We analyzed two experimental groups, namely ballet dancers and professional dancers of other styles, and a control group. A sample of 249 people took part in the study: 155 women and 94 men aged 18–56 years. Participants filled in The Imagination in Sport Questionnaire and Polish adaptations of the Big Five Inventory—Short and the Body Esteem Scale. Results indicated that while each experimental group differed significantly from the control group in terms of their imagery ability, there were no differences between the two experimental groups. Findings revealed that personality traits, mainly higher openness to experience, and body esteem, mainly related to physical condition, were significant predictors of higher imagery ability in all groups.

## 1. Introduction

Dancing is a performing art which merges art and sport as it is related to the expression of self, which is a highly emotional experience [1], as well as the integration of movement and cognition [2]. Therefore, imagery programs have been shown to improve the quality of dancers’ performance [3,4,5]. Training with imagery, in which the individual imagines the movements, improves movement quality and allows for more effective memorization of choreography. More specifically, by using imagery, the dancer can strengthen the movement patterns, helping with skill acquisition and refinement and detail retention [6,7,8].

Professional dancers can improve their ability to perform complex and complicated movements through what is referred to as “marking” or making movements in a very slow way without using full physical strength and energy. In this way, they learn motion not only through its physical but also mental reproduction. Thanks to this, they improve their technique while saving strength and energy by mainly using imagery of motion [9]. Imagery is an inseparable element of mental training, which has proven its effectiveness in numerous studies [10]. Thus, the incorporation of cognitive imagery training into “deliberate” practice has been promoted, and multiple studies [11,12,13,14] have established the positive influence of imagery on sport (i.e., self-confidence and reduced anxiety).

Imagery is a complex and broadly defined concept that can be used as the general ability in an individual’s daily life routine which drives one’s behavior; however, it can be specifically used for performance optimization, emphasizing the situational aspects of performance. To address these concepts, Budnik-Przybylska [15] developed a questionnaire (The Imagination in Sport Questionnaire) specifically devoted to the assessment of these components of imagery in athletes. Thus, in the present study, both the tendency to use general imagery and situational (connected with performance) imagery will be analyzed.

There has been evidence that people who practice sport regularly present higher imagery ability than novices or nonathletes [16]. Still, there have been few studies on its impact on dancing [6], which is a unique activity which merges, as mentioned earlier, competitive performance similar to sports with artistic aspects. Therefore, it is worth examining whether dancers display higher abilities in imagery in comparison to nondancers. This is an understudied issue, since earlier studies on imagery in dance typically included small samples [17]. Moreover, studies investigating the predictors of imagery, including general ability, in dancers are lacking.

### 1.1. Predictors of Imagery in Dancers

Individual characteristics of a performer can influence and predict imagery [16,17,18]. This provides a starting point for researchers to develop testable predictions [18,19]. According to the revised model of deliberate imagery use [18,19] and the model of deliberate imagery use [20], dispositional factors such as personality traits or body esteem might be predictors of imagery. Specifically, Anuar et al. [21] found that emotional regulation predicts imagery ability. However, there are not many studies examining dispositional factors related to imagery.

Therefore, for the purposes of the current investigation, which put emphasis on personality traits, we have selected one of the most popular five-factor personality models [22]. In this personality model, openness to experience has been strongly connected to imagery [22,23] and higher performance in sport [24].

Openness to experience is also referred to as intellectual inquisitiveness or independence. It is understood as a bipolar dimension, with lower scores representing mental closure and low curiosity. According to Costa and McCrae [22,24], openness to experience is related to imagined experiences described as fantasies, as well as having a vivid and creative imagination. It is the plasticity of imagined experiences and the ability to create them that is crucial for good visualization effects involved in mental training [25], which is why this feature can affect the effectiveness of imagining. In addition, other components such as esthetic sensitivity, openness to other people’s emotional states, and actions that actively seek new stimuli, as well as intellectual curiosity, may be important in imagining, because to achieve efficient visualization, it is important to use as many senses as possible. Openness is especially needed at the very beginning of the creative process and is closely related to the ease of assimilation [26,27]. Openness, in terms of the five-factor personality theory, means a tendency to go beyond the framework of a chosen field, and the ease of processing and assimilating new information is intensified in people who are highly creative [22]. Openness to experience was also noticed among artists [28], as well as in creative people who have a higher demand for new stimuli and unusual experiences [29]. In dancers, the level of psychoticism was higher than in the control groups [30], which may have a relationship with predisposition for creative problem-solving and creativity as well as openness to experience [31]. With regard to creativity, there are studies in which it has been shown that a greater intensity of this feature is visible in dancers of styles such as contemporary dance than it is in classical or ballet dancers [32]. In the same studies, the higher openness to experience of dancers of all these styles was confirmed in comparison with the control group. Other specific features required from dancers are diligence, perseverance, discipline, regularity, and attaining perfection in performing specific tasks [33]. For example, conscientiousness increases the sensitivity to tempo change [34].

Besides the traits of personality, another crucial aspect of performance quality in dancing is body functioning. The dancer’s working tool is the body, which is why physical conditioning is very important in this profession. There are a number of psychophysical requirements that must be achieved by a person who develops a professional career in dance. Caring for the physical condition to maintain impeccable condition is a basic requirement. The dancer’s body must also perform some esthetic functions in addition to performing instrumental functions based on top-down-imposed standards. Regardless of the chosen style of dance, people who decide to compete must have physical strength [35]. People who regularly engage in physical effort, including professional athletes, reveal a more positive picture of their own body in studies than people with a sedentary lifestyle [36,37]. Physical exercise contributes to an increase in physical ability, and thus to a more positive image of one’s body [38]. Professional and amateur dancers positively perceive their body and more positively assess their physical fitness and condition and health [39].

Moreover, there are a number of studies on professional dancers, especially classical dancers, which show that many of them have problems with disorders related to the body and nutrition. The increased incidence of eating disorders in specific groups, such as dancers, athletes, and models, is caused by the high cultural and social requirements associated with these professions, but is also influenced by personality factors such as perfectionism, high self-imposed standards, and ambitions [40].

### 1.2. Study Aim

The first aim of the study was to analyze whether dancers, with a separate sample of ballet dancers, differ from the control group in terms of (general and situational) imagery ability. Differences between the two groups of dancers as well as group differences in personality traits and body esteem were also explored. Additionally, the effects of gender and age factors on imagery ability (general and situational) were also analyzed. Based on aforementioned previous findings, we hypothesized that participants with experience in dancing will display higher imagery ability (hypothesis 1) as well as openness to experience (hypothesis 2) in comparison with the control group. Additionally, due to the perfection and regularity required in ballet [33], we hypothesized that this type of dancers would obtain higher scores in consciousness (hypothesis 3). We also asked whether there would be group differences in body esteem.

Following the second study aim, personality traits and body image were tested as predictors of imagery ability (general and situational) in the three examined groups. We expected that openness to experience would be a predictor of imagery ability in all groups (hypothesis 4). Furthermore, we explored whether other dimensions of personality and body esteem would be predictors of imagery ability (general and situational).

## 2. Materials and Methods

### 2.1. Participants

In total, 249 people were examined, including 155 women (62.2%). The participants were aged between 18 and 56 years (*M* = 25.07, *SD* = 5.82), and were divided into three groups:

(1) The control group, consisting of 104 nondancers, with no professional or recreational experience in dancing (58.7% women; age: *M* = 23.52, *SD* = 3.77); 49% declared having completed secondary education and 49% higher education, 58% were married, and 89% were childless;

(2) Eighty-two people who had experience with dance but were not graduates of ballet schools (63.4% women; age: *M* = 25.44, *SD* = 6.50); 42% declared having completed secondary education and 54% higher education, 54% were married, and 85% were childless;

(3) Sixty-three people who had experience in dancing and were graduates of ballet schools (66.7% women; age: *M* = 27.16, *SD* = 6.94); 49% declared having completed secondary education and 43% higher education, 43% were married, and 79% were childless.

As women are overrepresented in dancing, which as an expressive activity, has been perceived as feminine [41], the majority of our participants are females. The number of women was balanced across the three different groups.

All subjects gave their informed consent for inclusion before their participation in the study. The study was conducted in accordance with the Declaration of Helsinki, and the Ethics Committee of University of Gdansk approved the protocol (11/2015) before the commencement of the study.

### 2.2. Procedure

The participants were volunteers who completed a set of questionnaires, for which they were assured anonymity. The selection for the examined groups was carried out with the contribution of a Masters student supervised by the first and second authors, who contacted the people connected with dancing at ballet schools, dance theatres, operas, or theatres where ballet groups existed (dancers and ballet dancers), as well as the nondancers (control group), and administered the questionnaires. Dancers and ballet dancers were recruited from the lists of graduates of ballet schools, a list of graduates of the Krakow School of Choreography—Dance Theatre Department in Bytom, a list of current ballet dancers of the national opera and dance theatres, a list of current and active dancers on the polskiepolska.pl portal, a list of dancers of the professional folklore band Mazowsze and Silesia, and groups on a social network intended for professional dancers.

### 2.3. Measurements

The participants completed the information form, The Imagination in Sport Questionnaire (the modified version: The Imagination in Action), the BFI-S Questionnaire, and the Body Esteem Scale.

*Information form.* The information form was used as an introduction to a set of tests examining specific aspects of this work. The form contained information on ensuring the anonymity of the person taking part in the study and a request for fairness and accurate completion of all questions. The information form mainly concerned sociodemographic aspects, such as age, gender, and questions related to specific aspects of their professional activity.

*Imagery.* The Imagination in Sport Questionnaire (ISQ) [15] is a multidimensional 51-item measurement tool that consists of seven subscales, i.e., physiological feelings (noticeable changes in body functioning), modalities (use of senses besides the visual sense), ease/control (ease and control of the imagined scene), perspective (juggling of different perspectives of the imagined scene), affirmations (positive attitude during competition), visual (visual sense), and general (general tendency to use imagery). The participants imagined a competitive situation for 60 s in as detailed and realistic a manner as possible. After this task was completed, the participants were instructed to respond to the 51 items and rate the different aspects of the image on a scale from 1 (not at all) to 5 (completely so). All subscales (except one that was named “general”) were related to the imagined situation: i.e., situational imagery. The “general” subscale consisted of six questions and was developed separately to assess the general tendency to use imagery: i.e., general imagery. The ISQ demonstrates good stability (test–retest reliability ranged from *r* = 0.55 to *r* = 0.74) over a 3-week period and sound internal consistency (indicated by Cronbach’s alpha, which ranged from 0.64 to 0.79). A confirmatory factor analysis indicated acceptable model fit indices for the ISQ’s seven-factor structure (NC (normed chi-square) = 2416.63, df (degree of freedom) = 1203, GFI (Goodness of fit index) = 0.944, AGFI (Adjusted Goodness of Fit index) = 0.944, RMSEA (Root Mean Square Error of Approximation) = 0.056).

For the purposes of this study, some items were changed (items 27–29, 31–40, and 45). The changes concerned details in adjusting items to specific aspects of performance instead of competitions. For example, item 32 in the original test—“Positively tuning for a successful start”— was changed to “Positively tuning for a successful performance”). In the current experiment, the ISQ showed better reliability (Cronbach’s alpha) for each subscale: feelings (0.84), modalities (0.69); ease control (0.89), perspective (0.84), affirmation (0.88), visual (0.75), and general (0.84).

In the following are some examples of items for each subscale: for feelings: “How clearly was the feeling of the movements executed by you?”; for modalities: “How clearly did you hear the sounds occurring in this situation?”; for ease control: “How easy is it to recall the episode from the end to the beginning?”; for perspective: “Can you at any time stop the movie and watch the ‘freeze frame’ in detail?”; for affirmation: “You feel confident during the competitions”; for visual: “Were colors that occurred in this situation clear?”; for general: “Do you imagine the events waiting for you?”.

*Personality.* In order to study personality, the Polish version [42] of the Big Five Inventory—Short (BFI-S) was used [43]. It is a 15-item tool with a seven-point scale of answers, where 1 means definitely not and 7 definitely yes. This tool is used to measure personality in terms of a five-factor personality theory. Specific subscales measure the following traits: extraversion, openness to experience, agreeableness, neuroticism, and conscientiousness. Due to its short form, this scale is increasingly being used in exploratory research measuring many variables [44,45]. The reliability of the subscales are as follows: for extraversion, Cronbach’s alpha = 0.62; for openness to experience, Cronbach’s alpha = 0.73; for agreeableness, Cronbach’s alpha = 0.50; for neuroticism, Cronbach’s alpha = 0.57; and for conscientiousness, Cronbach’s alpha = 0.67 [43]. Example of items: *I perceived myself as someone who: is talkative* (extraversion); *is original and ingenious* (openness to experience); *forgives easily* (agreeableness); *worries a lot* (neuroticism); *is lazy* (conscientiousness).

The Polish adaptation [46] of the Body Esteem Scale originally developed by Franzoi and Shields [47] is a 35-point tool with a five-point scale, where 1 means I have strong negative feelings and 5 means I have strong positive feelings. Women’s subscales are: physical condition, referring to parameters related to endurance, strength, and agility; weight control, defining the ratio of appetite and assessment of the part of the body that may be subject to possible modification through the use of an appropriate diet or increased physical activity; and sexual attractiveness, defining the level of satisfaction with the appearance of particular parts of the body directly related to sexuality. The subscales for men are: body strength, physical attractiveness, and physical condition. Reliability of subscales was high both for females (with Cronbach’s alpha ranging from 0.80 to 0.89) and males (with Cronbach’s alpha ranging from 0.85 to 0.88. The coefficients of reliability for female subscales are, respectively: for sexual attractiveness, Cronbach’s alpha = 0.80; for weight concern, Cronbach’s alpha = 0.89; and for physical condition, Cronbach’s alpha = 0.82. For male subscales, the coefficients of reliability are, respectively: for physical attractiveness, Cronbach’s alpha = 0.85; for upper body strength, Cronbach’s alpha = 0.85; and for physical condition, Cronbach’s alpha = 0.88 [46].

Example of items for women: *physical stamina, reflexes* (physical condition); *appetite, waist* (weight control); *lips, breasts* (sexual attractiveness). Example of items for men: *muscular strength, biceps* (body strength); *nose, lips* (physical attractiveness); *physical stamina, energy level* (physical condition).

### 2.4. Statistical Analysis

Average and standard deviations of the variables tested were calculated. For age effect, the Spearman correlation was used, and for gender effect, UNIANOVA was applied. For direct group comparisons, a one-way analysis of variance (ANOVA) was used. Post-hoc Tukey test was applied to compare the three different groups. In order to determine the predictors of the imagery ability, linear regression analyses were used. The significance level was set at α = 0.05. The IBM SPSS Statistics version 25 (IBM, Armonk, New York, USA) was used to perform the calculations.

## 3. Results

### 3.1. Gender and Age Effects on Imagery Ability (General and Situational)

Before testing the hypothesis, we examined the age and gender effects on imagery ability (general and situational). Spearman’s correlation analysis revealed no association between age and general and situation imagery (for general imagery: rho = −0.113, *p* = n.s.; for situational imagery: rho = 0.060, *p* = n.s.). For general imagery, the main effect of gender was insignificant (F(1; 248) = 0.186; *p* = 0.667; η^2^ = 0.001), but the main effect of the group was significant (F(2; 248) = 3.228; *p* = 0.041; η^2^ = 0.026), and no interaction between those two factors occurred (F(2; 248) = 0.099; *p* = 0.906; η^2^ = 0.01). For situational imagery, the main effect of gender was insignificant (F(1; 248) = 2.707; *p* = 0.101; η^2^ = 0.011), but the main effect of the group was significant (F(2; 248) = 19.569; *p* < 0.001; η^2^ = 0.139), and no interaction between those two factors occurred (F(2; 248) = 1.960; *p* = 0.143; η^2^ = 0.016).

### 3.2. Group Differences in Imagery Ability (General and Situational), Personality, and Body Esteem

Next, we sought to answer hypotheses and questions formulated according with the first study aim. Thus, we tested hypotheses 1–3. We also checked the group effect on body esteem. A one-way between-subjects ANOVA was conducted to compare the effect of groups on the five dimensions of personality as well as for imagery and body esteem. Results are shown in Table 1.

Our results indicated that while both groups of dancers differed from the control group in imagery (situational; ballet dancers did not differ from the control group in general imagery), personality and body esteem levels showed a more varied trend. Specifically, both groups of dancers achieved higher scores in openness to experience, but for ballet dancers, also in conscientiousness. Both groups of dancers also achieved higher scores in all imagery scales. They perceived their attractiveness and physical condition to be higher. Ballet dancers were also more attentive to their weight (women) or upper body strength (men).

According to the second study aim, we sought predictors of imagery ability in the three examined groups. The obtained results are presented below.

### 3.3. Personality Dimensions as Predictors of Imagery Ability (General and Situational)

To test whether openness to experience (hypothesis 4) and other personality dimensions would be predictors of imagery ability, we conducted linear regression analysis.

Regression analysis revealed that personality explained general imagery ability in 41% (*R*^2^ = 0.41, *R* = 0.64, Corrected *R* = 0.38, *F(5,98)* = 13.482, *p* < 0.001) of the control group, 49% of dancers (*R*^2^ = 0.49, *R* = 0.70, Corrected *R* = 0.45, *F(5,76)* = 14.36, *p* < 0.001), and 67% of ballet dancers (*R*^2^ = 0.67, *R* = 0.82, Corrected *R* = 0.64, *F(5,57)* = 23.13, *p* < 0.001).

Specifically, the contribution of each subscale of personality to the explanation of general imagery as the dependent variable revealed the following predictors: openness to experiences for all groups (control group *β* = 0.56, *t* = 6.959, *p* < 0.001, dancers *β* = 0.59, *t* = 7.03, *p* < 0.001, ballet dancers *β* = 0.69, *t* = 6.85, *p<* 0.001), extraversion in the control group (*β* = 0.17, *t* = 2.09, *p* < 0.05), and conscientiousness in the group of dancers (*β* = 0.28, *t* = 3.21, *p* < 0.01);

Regarding the variance in situational imagery ability of the different groups, personality explained 31% of the total variance (*R*^2^ = 0.31, *R* = 0.56, Corrected *R* = 0.27, *F(5,98)* = 8.72, *p* < 0.001) in the control group, 23% in the group of dancers (*R*^2^ = 0.23, *R* = 0.48, Corrected *R* = 0.18, *F(5,76)* = 4.56, *p* < 0.001), and 59% in the group of ballet dancers (*R*^2^ = 0.31, *R* = 0.77, Corrected *R* = 0.55, *F(5,57)* = 16.37, *p* < 0.001).

The two regression analyses with situational imagery as the dependent variable revealed the following predictors: (1) personality: openness to experience was the most significant predictor in all groups: (*β* = 0.55, *t* = 6.38, *p* < 0.001) for the control group, (*β* = 0.47, *t* = 4.58, *p* < 0.001) for dancers, and (*β* = 0.40, *t* = 3.55, *p* < 0.001) for ballet dancers. Additionally, in the group of ballet dancers, the most significant predictor was conscientiousness (*β* = 0.34, *t* = 3.14, *p* < 0.001).

### 3.4. Body Esteem as a Predictor of Imagery (General and Situational)

Next, we analyzed if body esteem is the predictor of imagery ability. General imagery was not significant in the control group; however, it explained 17% of the total variance in the group of dancers (*R*^2^ = 0.17, *R* = 0.41, Corrected *R* = 0.14, *F(3,78)* = 5.27, *p* < 0.01) and 23% in ballet dancers (*R*^2^ = 0.23, *R* = 0.48, Corrected *R* = 0.19, *F(3,59)* = 5.89, *p* < 0.001).

In the following, we report the contribution of each subscale of body esteem to general imagery: physical condition for all groups (control group (*β* = 0.28, *t* = 1.99, *p* < 0.05), dancers (*β* = 0.34, *t* = 2.24, *p* < 0.05), ballet dancers (*β* = 0.42, *t* = 2.09, *p* < 0.05)); attractiveness *only* in dancers (*β* = 0.29, *t* = 2.30, *p* < 0.05) and weight control (*β* = −0.31, *t* = −2.13, *p* < 0.05).

Furthermore, body esteem affects situational imagery ability for 16% (*R*^2^ = 0.16, *R* = 0.40, Corrected *R* = 0.13, *F(3,100)* = 6.30, *p* < 0.01) of the control group, 12% of the group of dancers (*R*^2^ = 0.12, *R* = 0.34, Corrected *R* = 0.09, *F(3,78)* = 3.42, *p* < 0.05), and 18% of ballet dancers (*R*^2^ = 0.18, *R* = 0.43, Corrected *R* = 0.14, *F(3,59)* = 4.41, *p* < 0.01).

With regards to body esteem, the physical condition was the significant predictor in all groups: control (*β* = 0.396, *t* = 3.038, *p* < 0.01), dancers (*β* = 0.470, *t* = 3.024, *p* < 0.01), and ballet dancers (*β* = 0.521, *t* = 2.508, *p* < 0.05). Additionally, attractiveness (*β* = 0.235, *t* = 2.115, *p* < 0.05) and weight control (*β* = −0.420, *t* = −3.252, *p* < 0.01) were significant predictors in the control group.

## 4. Discussion

The first aim of the study was to compare dancers and ballet dancer with nondancers in regard to imagery ability (general and situational). Our findings suggest that imagery ability was higher in dancers, including ballet dancers, compared to the control group. Only in general imagery ability did the group of ballet dancers not differ from the control group. We hypothesized that dancers would report higher general imagery ability (hypothesis 1), and this assumption was confirmed, since ballet dancers had an average score of 4.21, dancers had an average score of 4.28, and the control group showed lower values (4.02). The subscales of situational imagery, which is associated with imagining the performance, confirmed this trend (hypothesis 1). This specific type of imagery might be the consequence of previous experiences and training in both studied groups. This is in line with previous studies on athletes, including elite athletes, who reported higher imagery ability [15,16,48,49,50] compared to novices and nonathletes. Therefore, imagery is a skill that improves with practice [51], and elite athletes use it more frequently and deliberately [52]. However, studies on dancers are scarce [6], and our study expands the knowledge concerning the role of imagery in this specific group of performers.

Our findings confirmed that dance and imagery are closely intertwined. During learning different sequences of movements, people start to use their kinesthetic imagery to become more accustomed to the feeling of the movements [53]. Taking an individual difference perspective, our study shows that dancers (from various dancing domains) might be more skilled in imagery in real life, even outside the domain of dance.

In the first study aim, we also compared dancers and ballet dancer with nondancers regarding personality traits and body esteem. Both groups of dancers displayed higher levels of openness to experience in comparison with nondancers. The second hypothesis related to the openness to experience of the dancers was thus confirmed. Higher openness to experience is also in line with results of previous research concerning high-risk sports [54] and dancing [55]. In the latter study, authors showed that modern/contemporary and jazz/musical dancers were more open to experience than ballet dancers; however, in their study, a control group was not present. In our study, both groups of dancers differed from nondancers and dancers achieved higher results in openness to experience than ballet dancers, although the latter were not significantly different.

Additionally, ballet dancers were more conscientious than nondancers. Dancing is a very demanding discipline which is connected with regular practice and striving for perfection [56,57]. Therefore, regarding hypothesis 3, the higher scores obtained by both groups of dancers compared to the control group in the conscientiousness subscale is not surprising. This result is also concurrent with the study conducted by Kaźmierczak et al. [58], in which athletes presented higher scores compared to less-active counterparts. That result was also confirmed in the study of Fink and Woschniak [55], where ballet and jazz/musical dancers displayed higher scores in conscientiousness compared to modern/contemporary dancers. It is understood as being a consequence of ballet’s specificity, i.e., strong adherence to predefined scripts or choreographies, which may be connected with more conventional or traditional ways of thinking. Furthermore, it has also been indicated that the training workload of ballet dancers is particularly high [35]. Hence, our third hypothesis was confirmed.

Additional analyses regarding body esteem showed that dancers (both groups) perceived their attractiveness and physical condition to be higher, but at the same time, they were more concerned about their weight. Ballet dancers obtained higher scores in the weight control subscale (women) or upper body strength (men) subscale than the control group. However, higher attention to weight is characteristic of all sports where weight is important [59,60,61]. Our findings are in line with the study conducted by Lewis and Scannell [62], who showed that subjects experienced in creative dance movement were more satisfied with their appearance, fitness, and body parts than subjects with less than five years of experience. Higher level of perceived attractiveness and physical condition is in accordance also with the study concerning physical exercise contribution and positive body image in general [38]. Our findings contradict the conclusions highlighted by Jauregui-Lobera [40] about negative body esteem among dancers.

As for the second study aim, we confirmed that personality and body esteem were significant predictors of imagery ability. As mentioned in the introduction, this is the understudied issue, especially regarding the influence of personality factors and, referring to the uniqueness of dancing, body esteem. Our results indicated that some personality traits, but not body esteem, were predictors of general imagery in all samples. Specifically, personality as a predictor of general imagery ability as well as of situational ability was significant in all analyzed groups, but was the most significant in ballet dancers (67% general imagery ability, 59% situational imagery ability). Thus, that high amount of variance in imagery (both general and situational) might have not been explained in the previous studies and should not be underestimated. The most important predictor of situational as well as general imagery ability was openness to experience. People who assimilate new information in different ways and are open to new ideas present higher imagery ability. Moreover, using imagination is most effective for people who have an innate predisposition [20]. Therefore, also the fourth hypothesis was confirmed.

The second significant personality trait predicting imagery ability was consciousness. However, for general imagery ability, it was a significant predictor in the groups of dancers, but when situational components were taken into account, consciousness was a significant predictor only in ballet dancers. It may be explained, as mentioned before, by the specific situation of imagery script. For ballet dancers, performance is very strict and movements should be more perfect than for other groups of dancers, whereas in general, the usage of imagery seems to be more useful for the group of dancers.

As far as body esteem is concerned, it was the predictor of situational imagery in all samples. Specifically, taking care of physical condition was the predictor of imagery, both general and situational, in all samples. Drawing on Hardy and Callow [53], we might hypothesize that imagery can support the connection between physical condition and movement learning and execution. Focusing on physical condition might facilitate imagery, regardless of experience in dance and in everyday life. Another result worth noticing is that weight control was a significant but negative predictor of imagery ability. People who are more focused on their weight might have problems with imagery because they are overwhelmed with cognitive processes. Such a result is crucial for ballet dancers, among whom a focus on weight control (the highest of all samples) might hinder imagery.

Therefore, body esteem might affect imagery, but this relation only occurs in the specific context when competitive situations are imagined. In this respect, personality traits, with particular emphasis on openness to experience, should be defined as universal predictors of imagery, both general and situational, regardless of personal experience in competing.

Concluding, we would like to highlight limitations of our study. There were discrepancies in the number of participants in the three groups. Indeed, in the future, the number of participants in specific age groups should be balanced.

In future research, larger samples and with more male participants should be included. More measures of imagery ability as well as personality measures of less general (than those from the five-factor model) traits should be used to better explain the specificity of dancers’ functioning. Moreover, other dispositional measures such as temperament, other concepts of personality, or mental toughness could be investigated as dispositional factors predicting imagery ability, in particular investigating the link between imagery and mental toughness [63]. Furthermore, in the future it would be worthwhile to link results of the Imagination in Sport Questionnaire with The Dance Imagery Questionnaire [12], which is a 16-item questionnaire measuring four different types of dancers’ imagery: technique imagery, role and movement quality imagery, mastery imagery, and goal imagery.

The present paper has a lot of relevant practical implications, not only for dance as a sport, but also for everyday life. Educators and coaches should be aware of predictors of imagery ability, which in turn could affect final dancers’ performance and mental training programs. Individual factors such as personality traits and body esteem should be taken into account when imagery ability is trained and developed. Our results expand the knowledge about individual differences among dancers and nondancers, especially regarding imagery ability, which might be implemented in interventions and training programs. Further, as we focused on personal factors (not sociocultural factors), our results might be viable cross-culturally.

## Figures and Tables

**Table 1:** Imagery personality and body esteem among control group dancers and ballet dancers.

	Control Group: N = 104		Dancers: N = 82		Ballet Dancers: N = 63		F Statistic df(2,246)		Eta-Square	Power	Tukey Test		
	*M*	*SD*	*M*	*SD*	*M*	*SD*	*F*	*p*			*1vs2*	*1vs3*	*2vs3*
IMAGERY													
General	4.02	0.69	4.28	0.59	4.21	0.83	3.34	0.04	0.03	0.63	0.04	ns	ns
Physiological feelings	3.32	0.87	3.84	0.84	3.67	1.00	8.18	0.00	0.06	0.96	0.00	0.04	ns
Modalities	2.59	0.73	2.89	0.76	2.86	0.83	4.12	0.02	0.03	0.73	0.03	0.07	ns
Ease control	3.47	0.77	3.96	0.66	4.01	0.83	14.13	0.00	0.10	1.00	0.00	0.00	ns
Perspective	3.10	0.86	3.55	0.74	3.60	0.88	9.74	0.00	0.07	0.98	0.00	0.00	ns
Affirmations	3.64	0.88	4.22	0.64	4.14	0.82	14.19	0.00	0.10	1.00	0.00	0.00	ns
Visual	3.30	0.71	3.82	0.74	3.72	0.87	11.97	0.00	0.09	1.00	0.00	0.00	ns
BFI-S													
Openness to experience Experience	5.23	1.10	5.94	0.80	5.77	1.20	12.04	0.00	0.09	1.00	0.00	0.00	ns
Conscientiousness	5.04	0.99	5.33	0.92	5.54	1.05	5.20	0.01	0.04	0.83	ns	0.01	ns
Extraversion	4.26	1.15	4.36	1.13	4.06	1.27	1.18	0.31	0.01	0.26	ns	ns	ns
Agreeableness	4.72	1.05	4.85	1.06	4.93	1.21	0.79	0.45	0.01	0.19	ns	ns	ns
Neuroticism	4.33	1.18	4.20	1.26	4.13	1.15	0.61	0.55	0.01	0.15	ns	ns	ns
BODY ESTEEM													
Attractiveness	3.63	0.46	3.84	0.53	3.84	0.71	4.16	0.02	0.03	0.73	0.04	0.05	ns
Weight control	3.44	0.69	3.62	0.68	3.77	0.86	4.05	0.02	0.03	0.72	ns	0.02	ns
Phys. condition	3.52	0.63	3.85	0.56	3.96	0.72	11.86	0.001	0.09	0.99	0.00	0.00	ns

**Note**. 1vs2 = nondancers vs dancers; 1vs3 = nondancers vs ballet dancers; 2vs3 = dancers vs ballet dancers. BFI-S: Big Five Inventory—Short.
